# taxize: taxonomic search and retrieval in R

**DOI:** 10.12688/f1000research.2-191.v2

**Published:** 2013-10-28

**Authors:** Scott A. Chamberlain, Eduard Szöcs

**Affiliations:** 1 Biology, Simon Fraser University, Burnaby, Canada; 2 Institute for Environmental Sciences, University Koblenz-Landau, Landau, Germany

## Abstract

All species are hierarchically related to one another, and we use taxonomic names to label the nodes in this hierarchy. Taxonomic data is becoming increasingly available on the web, but scientists need a way to access it in a programmatic fashion that’s easy and reproducible. We have developed taxize, an open-source software package (freely available from http://cran.r-project.org/web/packages/taxize/index.html) for the R language. taxize provides simple, programmatic access to taxonomic data for 13 data sources around the web. We discuss the need for a taxonomic toolbelt in R, and outline a suite of use cases for which taxize is ideally suited (including a full workflow as an appendix). The taxize package facilitates open and reproducible science by allowing taxonomic data collection to be done in the open-source R platform.

## Introduction

Evolution by natural selection has led to a hierarchical relationship among all living organisms. Thus, species are categorized using a taxonomic hierarchy, starting with the binomial species name (e.g, *Homo sapiens*), moving up to genus ( *Homo*), then family ( *Hominidae*), and on up to Domain ( *Eukarya*). Although taxonomic classifications are human constructs created to understand the real phylogeny of life ^1^, they are nonetheless essential to organize the vast diversity of organisms. Biologists, whether studying organisms at the cell, organismal, or community level, can put their study objects into taxonomic context, allowing them to infer close and distant relatives, find relevant literature, and more.

The use of taxonomic names is, unfortunately, not straightforward. Taxonomic names often vary due to name revisions at the generic or specific levels, lumping or splitting lower taxa (genera, species) among higher taxa (families), and name spelling changes. For example, a study found that a compilation of 308,000 plant observations from 51 digitized herbarium records had 22,100 unique taxon names, of which only 13,000 were accepted names ^2, 3^. In addition, there is no one authoritative source of taxonomic names for all taxa - although, there are taxon specific sources that are used by many scientists. Different sources (e.g., uBio [Universal Biological Indexer and Organizer], Tropicos, ITIS [Integrated Taxonomic Information Service]) may use different accepted names for the same taxon. For example, while ITIS has *Helianthus x glaucus* as an accepted name, The Plant List ( http://www.theplantlist.org) gives that name as unresolved. But *Helianthus glaucus* is an accepted name in The Plant List, while ITIS does not list this name.

One attempt to help inconsistencies in taxonomy is the use of numeric codes. For example, ITIS assigns a Taxonomic Serial Number (TSN) to each taxon, while uBio assigns each taxon a NameBank identifier (namebankID), and Tropicos assigns their own identifier to each taxon. Codes are helpful within a database as they can easily refer to, for example, *Helianthus annuus* with a code like 123456 instead of its whole name. However, each database uses their own code; in this case for *Helianthus annuus*, ITIS uses 36616, uBio uses 2658020, and Tropicos uses 40022652. As there are no universal codes for taxa across databases, this can lead to additional confusion. Last, name comparisons across databases have to be done with the actual names, not the codes.

Taxonomic data is getting easier to obtain through the web (e.g., http://eol.org/). However, there are a number of good reasons to obtain taxonomic information programatically rather than through a web interface. First, if you have more than a few names to look up on a website, it can take quite a long time to enter each name, get data, and repeat for each species. Programatically getting taxonomic names solves the problem by looping over a list of names. In addition, doing taxonomic searching, etc. becomes reproducible. With increasing reports of irreproducibility in science ^4, 5^, it is extremely important to make science workflows repeatable.

The R language is widely used by biologists, and now has over 5,000 packages on the Comprehensive R Archive Network (CRAN) to extend R. R is great for manipulating, visualizing and fitting statistical models to data. Gentleman *et al.* ^6^ give a detailed discussion of advantages of R in computational biology. Getting data from the web will be increasingly common as more and more data gets moved to the cloud. Therefore, there is a need to get data from the web directly into R. Increasingly, data is available from the web via application programming interfaces (API). These allow computers to talk to one another using code that is not human readable, but is machine readable. Web APIs often define a number of methods that allow users to search for a species name, or retrieve the synonyms for a species name, for example. A further advantage of APIs is that they are language agnostic, meaning that data can be consumed in almost any computing context, allowing users to interact with the web API without having to know the details of the code. Moreover data can be accessed from every computer, whereas for example an Excel file can only be opened in a few programs.

The goal of taxize is to make many use cases that involve retrieving and resolving taxonomic names easy and reproducible. In taxize, we have written a suite of R functions that interact with many taxonomic data sources via their web APIs ( Table 1). The interface to each function is usually a simple list of species names, just as a user would enter when interacting with a website. Therefore, we hope that moving from a web to an R interface for taxonomic names will be relatively seamless (if one is already nominally familiar with R).

**Table 1.** Some key functions in taxize, what they do, and their data sources.

Function name	What it does	Source
apg_lookup	Changes names to match the APGIII list	Angiosperm Phylogeny Group http://www.mobot.org/MOBOT/research/APweb/
classification	Upstream classification	Various
col_children	Direct children	Catalogue of Life http://www.catalogueoflife.org/
col_downstream	Downstream taxa to specified rank	Catalogue of Life http://www.catalogueoflife.org/
eol_hierarchy	Upstream classification	Encyclopedia of Life http://eol.org/
eol_search	Search EOL taxon information	Encyclopedia of Life http://eol.org/
get_seqs	Get NCBI sequences	National Center for Biotechnology Information ^7^
get_tsn	Get ITIS TSN	Integrated Taxonomic Information System http://www.itis.gov/
get_uid	Get NCBI UID	National Center for Biotechnology Information ^7^
gisd_isinvasive	Invasiveness status	Global Invasive Species Database http://www.issg.org/database/welcome/
gni_parse	Parse scientific names into components	Global Names Index http://gni.globalnames.org/
gni_search	Search EOL’s global names index	Global Names Index http://gni.globalnames.org/
gnr_resolve	Resolve names using EOL’s global names index	Global Names Resolver http://resolver.globalnames.org/
itis_downstream	Downstream taxa to specified rank	Integrated Taxonomic Information System http://www.itis.gov/
iucn_status	IUCN status	IUCN Red List http://www.iucnredlist.org
phylomatic_tree	Get a plant Phylogeny	Phylomatic ^8^
plantminer	Search Plantminer	Plantminer ^9^
searchbycommonname	Search ITIS by common name	Integrated Taxonomic Information System http://www.itis.gov/
searchbyscientificname	Search ITIS by scientific name	Integrated Taxonomic Information System http://www.itis.gov/
tax_name	Get taxonomic name for specific rank	Various
tax_rank	Get rank of a taxonomic name	Various
tnrs	Resolve names using iPlant	iPlant Taxonomic Name Resolution Service http://tnrs.iplantcollaborative.org/
tp_acceptednames	Check for accepted names using Tropicos	Tropicos http://www.tropicos.org/
tpl_search	Search the Plant List	The Plant List http://www.theplantlist.org
ubio_namebank	Search uBio	uBio http://www.ubio.org/index.php?pagename=sample_tools

Here, we justify the need for programmatic taxonomic resolution tools like taxize, discuss our data sources, and run through a suite of use cases to demonstrate the variety of ways that users can use taxize.

## Why do we need taxize?

There is a large suite of applications developed around the problem of searching for, resolving, and getting higher taxonomy for species names. For example, Linnaeus http://linnaeus.sourceforge.net/ provides the ability to search for taxonomic names in documents and normalize those names found. In addition, there are many web interfaces to search for and normalize names such as Encyclopedia of Life’s Global Names Resolver http://resolver.globalnames.org/, uBio tools http://www.ubio.org/index.php?pagename=sample_tools, and iPlant’s Taxonomic Name Resolution Service http://tnrs.iplantcollaborative.org/.

All of these data repositories provide ways to search for taxonomic names and resolve them in some cases. However, scientists ideally need a tool that is free and can be used programmatically, thereby facilitating reproducible research. The goal of taxize is to facilitate the creation of reproducible and easy to use workflows for searching for taxonomic names, resolving them, getting higher taxonomic names, and other tasks related to research dealing with species.

## Data sources and package details

taxize uses many data sources ( Table 1), and more can be easily added. There are two common tasks provided by the data sources: name search and name resolution. Other functionality in taxize includes retrieving a classification tree for a species, or retrieving child taxa of a focal taxon. One of the data sources (Phylomatic) returns phylogenies, while another (NCBI) returns genetic sequence data. However, there are other R packages that are focused solely on sequence data, such as rsnps ^10^, rentrez ^11^, BoSSA ^12^, and ape ^13^, so taxize does not venture deeply into these other domains.

Some of the data sources taxize interacts with require authentication. That is, in addition to the search terms the user provides (e.g., *Homo sapiens*), the data provider requires an alphanumeric identification key. This is necessary in some cases so that API providers can 1) better prevent databases crashing from too many requests, 2) collect analytics on requests to their API to provide better performance, etc., and 3) provide user level modification of rules for interacting with the API. The services that require an API key in taxize are: Encyclopedia of Life (EOL) http://eol.org/, the Universal Biological Indexer and Organizer (uBio) http://www.ubio.org/index.php?pagename=sample_tools, Tropicos http://www.tropicos.org/, and Plantminer ^9^. One can easily obtain API keys by visiting the website of each service (see Table 1 for links to each site). There are two typical ways of using API keys. First, you can pass in your API key in a function call (e.g., *ubio_namebank(srchName=’Ursus americanus’, key=’your_alphanumeric_key’)*). Second, you can store your key in the .Rprofile file, which is a common place to store settings. We recommend the second option as it simplifies function calls as taxize detects the stored keys.

taxize would not have been possible without the work of others. taxize uses httr ^14^ and RCurl ^15^ for performing calls to web APIs, XML ^16^ for parsing XML, RJSONIO ^17^ for parsing JSON, and stringr ^18^ and plyr ^19^ for manipulating data.

New data sources can be added; for example, we plan to add the following sources: Wikispecies and The Tree of Life. A connection to www.freshwaterecology.info (a database with autecological characteristics, ecological preferences and biological traits as well as distribution patterns of more than 12,000 European freshwater organisms belonging to fish, macro-invertebrates, macrophytes, diatoms and phytoplankton) will be finished when their new API is released. In addition, the authors welcome further suggestions of data sources to be added.

## Use cases

### First, install taxize

First, one must install and load taxize into the R session.



                        install.packages
                        (
                        "taxize"
                        )

                        library
                        (taxize)           
                    


Advanced users can also download and install the latest development copy from GitHub https://github. com/ropensci/taxize_, also permanently available at http://dx.doi.org/10.5281/zenodo.7097.

### Resolve taxonomic names

This is a common task in biology. We often have a list of species names and we want to know a) if we have the most up to date names, b) if our names are spelled correctly, and c) the scientific name for a common name. One way to resolve names is via the Global Names Resolver (GNR) service provided by the Encyclopedia of Life http://resolver.globalnames.org/. Here, on can search for two misspelled names:



                        temp 
                        <- gnr_resolve
                        (
                        names
                         = 
                        c
                        (
                        "Helianthos annus"
                        ٫       

                                                       
                        "Homo saapiens"
                        ))        

                        temp[ ٫ -
                        c
                        (
                        1
                        ٫
                        4
                        )]                                        

                                                                                
#                     ched_name       data_source_title 
# 1        Helianthus annuus L.       Catalogue of Life 
# 2            Helianthus annus GBIF Taxonomic Backbone 
# 3            Helianthus annus                     EOL 
# 4         Helianthus annus L.                     EOL 
# 5            Helianthus annus           uBio NameBank 
# 6 Homo sapiens Linnaeus٫ 1758       Catalogue of Life 
                    


The correct spellings are *Helianthus annuus* and *Homo sapiens*. Another approach uses the Taxonomic Name Resolution Service via the Taxosaurus API http:// taxosaurus.org/ developed by iPLant and the Phylotastic organization. In this example is a list of species names, some of which are misspelled, and then call the API with the *tnrs* function.



                        mynames 
                        <- c
                        (
                        "Helianthus annuus"
                        ٫ 
                        "Pinus contort"
                        ٫             

                                     "Poa anua"
                        ٫
                        "Abis magnifica"
                        ٫ 
                        "Rosa california"
                        ٫   

                                     "Festuca arundinace"
                        ٫
                        "Sorbus occidentalos"
                        ٫       

                                     "Madia sateva"
                        )                                   

                        tnrs
                        (
                        query
                         = mynames) [ ٫ -
                        c
                        (
                        5
                        :
                        7
                        )]                             

                                                                                       
#         submittedName         acceptedName     sourceIdscore 
# 9   Helianthus annuus     Helianthus annus  iPlant_TNRS    1 
# 10  Helianthus annuus     Helianthus annus         NCBI    1 
# 4       Pinus contort       Pinus contorta  iPlant_TNRS 0.98 
# 5            Poa anua            Poa annua  iPlant_TNRS 0.96 
# 3      Abis magnifica      Abies magnifica  iPlant_TNRS 0.96 
# 7     Rosa california     Rosa californica  iPlant_TNRS 0.99 
# 8     Rosa california           California         NCBI    1 
# 2  Festuca arundinace  Festuca arundinacea  iPlant_TNRS 0.99 
# 1 Sorbus occidentalos  Sorbus occidentalis  iPlant_TNRS 0.99 
# 6        Madia sateva         Madia sativa  iPlant_TNRS 0.97 

                    


It turns out there are a few corrections: e.g., *Madia sateva* should be *Madia sativa*, and *Rosa california* should be *Rosa californica*. Note that this search worked because fuzzy matching was employed to retrieve names that were close, but not exact matches. Fuzzy matching is only available for plants in the TNRS service, so we advise using EOL’s Global Names Resolver if you need to resolve animal names.

taxize takes the approach that the user should be able to make decisions about what resource to trust, rather than making the decision on behalf of the user. Both the EOL GNR and the TNRS services provide data from a variety of data sources. The user may trust a specific data source, and thus may want to use the names from that data source. In the future, we may provide the ability for taxize to suggest the best match from a variety of sources.

Another common use case is when there are many synonyms for a species. In this example, there are six synonyms of the currently accepted name for a species.



                        library
                        (plyr)                                         
mynames 
                        <- c
                        (
                        "Helianthus annuus ssp. jaegeri"
                        ٫             

                                     "Helianthus annuus ssp. lenticularis"
                        ٫   

                                     "Helianthus annuus ssp. texanus"
                        ٫        

                                     "Helianthus annuus var. lenticularis"
                        ٫   

                                     "Helianthus annuus var. macrocarpus"
                        ٫    

                                     "Helianthus annuus var. texanus"
                        )        

                        tsn 
                        <- get_tsn
                        (mynames)                               

                        ldply
                        (tsn٫ itis_acceptname)                           

                                                                              
#   submittedTsn           acceptedName   acceptedTsn 
# 1       525928      Helianthus annuus         36616 
# 2       525929      Helianthus annuus         36616 
# 3       525930      Helianthus annuus         36616 
# 4       536095      Helianthus annuus         36616 
# 5       536096      Helianthus annuus         36616 
# 6       536097      Helianthus annuus         36616 

                    


### Retrieve higher taxonomic names

Another task biologists often face is getting higher taxonomic names for a taxa list. Having the higher taxonomy allows you to put into context the relationships of your species list. For example, you may find out that species A and species B are in Family C, which may lead to some interesting insight, as opposed to not knowing that Species A and B are closely related. This also makes it easy to aggregate/standardize data to a specific taxonomic level (e.g., family level) or to match data to other databases with different taxonomic resolution (e.g., trait databases).

A number of data sources in taxize provide the capability to retrieve higher taxonomic names, but we will highlight two of the more useful ones: Integrated Taxonomic Information System (ITIS) http://www.itis.gov/ and National Center for Biotechnology Information (NCBI) ^7^. First, search for two species, *Abies procera* and *Pinus contorta* within ITIS.



                        specieslist 
                        <- c
                        (
                        "Abies procera"
                        ٫ 
                        "Pinus contorta"
                        )  

                        classification
                        (specieslist٫ 
                        db
                         = 
                        "itis"
                        )             

                                                                             
# $`Abies procera`                                   
#         rankName           taxonName          tsn  
# 1        Kingdom             Plantae       202422  
# 2     Subkingdom      Viridaeplantae       846492  
# 3   Infrakingdom        Streptophyta       846494  
# 4       Division        Tracheophyta       846496  
# 5    Subdivision     Spermatophytina       846504  
# 6  Infradivision        Gymnospermae       846506  
# 7          Class           Pinopsida       500009  
# 8          Order             Pinales       500028  
# 9         Family            Pinaceae        18030  
# 10         Genus               Abies        18031  
# 11       Species       Abies procera       181835  

                                                                             
# $`Pinus contorta`                                  
#           rankName           taxonName         tsn 
# 1          Kingdom             Plantae      202422 
# 2       Subkingdom      Viridaeplantae      846492 
# 3     Infrakingdom        Streptophyta      846494 
# 4         Division        Tracheophyta      846496 
# 5      Subdivision     Spermatophytina      846504 
# 6    Infradivision        Gymnospermae      846506 
# 7            Class           Pinopsida      500009 
# 8            Order             Pinales      500028 
# 9           Family            Pinaceae       18030 
# 10           Genus               Pinus       18035 
# 11         Species      Pinus contorta      183327 
                    


It turns out both species are in the family Pinaceae. You can also get this type of information from the NCBI by excuting the following code in R: *classification(specieslist, db = ’ncbi’)*.

Instead of a full classification, you may only want a single name, say a family name for your species of interest. The function *tax_name* is built just for this purpose. As with the *classification*-function you can specify the data source with the *db* argument, either ITIS or NCBI.



                        tax_name
                        (
                        query
                         = 
                        "Helianthus annuus"
                        ٫ 
                        get
                         = 
                        "family"
                        ٫

                                 db
                         = 
                        "itis"
                        )                                

                                                                             
#       family                                       
# 1 Asteraceae                                       

                                                                                                                                     

                        tax_name
                        (
                        query
                         = 
                        "Helianthus annuus"
                        ٫ 
                        get
                         = 
                        "family"
                        ٫

                                 db
                         = 
                        "ncbi"
                        )                                

                                                                             
#       family                                       
# 1 Asteraceae                                       
                    


If a data source does not provide information on the queried species, the result could be taken from another source and the results from the different sources could be pooled.

### Interactive name selection

As mentioned previously most databases use a numeric code to reference a species. A general workflow in taxize is: Retrieve Code for the queried species and then use this code to query more data/information. Below are a few examples. When you run these examples in R, you are presented with a command prompt asking for the row that contains the name you would like back; that output is not printed below for brevity. In this example, the search term has many matches. The function returns a data.frame of the matches, and asks for the user to input which row number to accept.



                        get_tsn
                        (
                        searchterm
                         = 
                        "Heliastes"
                        ٫ 
                        searchtype
                         = 
                        "sciname"
                        )  

                                                                                  
#		 combinedname	   tsn                    
# 1	    Heliastes bicolor	615238                    
# 2	  Heliastes chrysurus	615250                    
# 3	    Heliastes cinctus	615573                    
# 4	 Heliastes dimidiatus	615257                    
# 5	 Heliastes hypsilepis	615273                    
# 6	Heliastes immaculatus	615639                    
# 7	Heliastes opercularis	615300                    
# 8	     Heliastes ovalis	615301                    
#  1                                                      
# NA                                                      
# attr(٫"class")                                          
# [1] "tsn"                                               

                    


In another example, you can pass in a long character vector of taxonomic names:



                        splist 
                        <- c
                        (
                        "annona cherimola"
                        ٫ 
                        'annona muricata'
                        ٫        

                                    "quercus robur"
                        ٫ 
                        "shorea robusta"
                        ٫           

                                    "pandanus patina"
                        ٫ 
                        "oryza sativa"
                        ٫           

                                    "durio zibethinus"
                        )                          

                        get_tsn
                        (
                        searchterm
                         = splist٫ 
                        searchtype
                         = 
                        "sciname"
                        )     

                                                                                 
# [1] "506198" "18098" "19405" "506787" "507376" "41976" 
# [7] "506099"                                           
# attr(٫"class")                                         
# [1] "tsn"                                              

                    


In another example, note that no match at all returns an NA:



                        get_uid
                        (
                        sciname 
                        = 
                        c
                        (
                        "Chironomus riparius"
                        ٫ 
                        "aaa vva"
                        ))

                                                                              
# [1] "315576" NA                                     
# attr(٫"class")                                      
# [1] "uid"                                           

                    


### Retrieve a phylogeny

**Figure 1.**
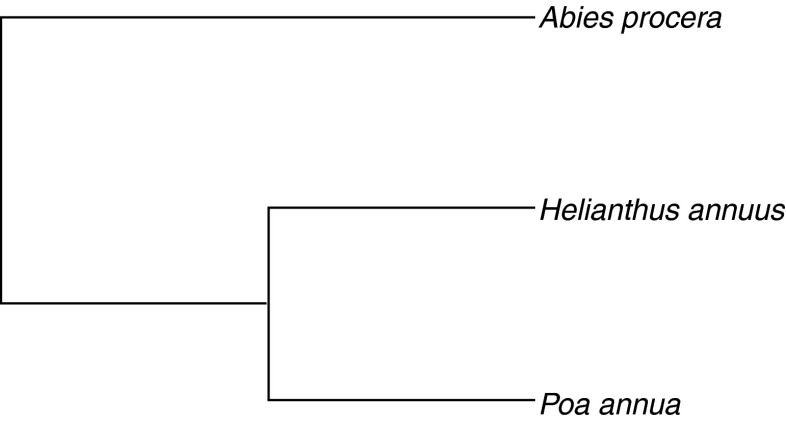
A phylogeny for three species. This phylogeny was produced using the *phylomatic_tree* function, which queries the Phylomatic database, and prunes a previously created phylogeny of plants.



Ecologists are increasingly taking a phylogenetic approach to ecology, applying phylogenies to topics such as the study of community structure ^20^, ecological networks ^21^, and functional trait ecology ^22^. Yet, many biologists are not adequately trained in reconstructing phylogenies. Fortunately, there are some sources for getting a phylogeny without having to know how to build one; one of these is for angiosperms, called Phylomatic ^8^. We have created a workflow in taxize that accepts a species list, and taxize works behind the scenes to get higher taxonomic names, which are required by Phylomatic to get a phylogeny. Here is a short example, producing the tree in Figure 1.

                        taxa 
                        <- c
                        (
                        "Poa annua"
                        ٫ 
                        "Abies procera"
                        ٫ 
                        "Helianthus annuus"
                        ) 

                        tree 
                        <- phylomatic_tree
                        (
                        taxa
                         = taxa)                         

                        tree$tip.label 
                        <- capwords
                        (tree$tip.label)                   

                        plot
                        (tree٫ 
                        cex
                         = 
                        1
                        )                                          
                    


Behind the scenes the function *phylomatic_tree* retrieves a Taxonomic Serial Number (TSN) from ITIS for each species name, then a string is created for each species like this *poaceae/oryza/oryza_sativa* (with format "family/genus/genus_epithet"). These strings are submitted to the Phylomatic API, and if no errors occur, a phylogeny in newick format is returned. The *phylomatic_tree()* function also cleans up the newick string and converts it to an ape *phylo* object, which can be used for plotting and phylogenetic analyses. Be aware that Phylomatic has certain limitations - refer to the paper describing Phylomatic ^8^ and the website http://phylodiversity.net/phylomatic/.

### What taxa are children of the taxon of interest?

If someone is not a taxonomic specialist on a particular taxon they probably do not know what children taxa are within a family, or within a genus. This task becomes especially unwieldy when there are a large number of taxa downstream. You can of course go to a website like Wikispecies http://species.wikimedia.org/wiki/Main_Page or Encyclopedia of Life http://eol.org/ to get downstream names. However, taxize provides an easy way to programatically search for downstream taxa, both for the Catalogue of Life (CoL) http://www.catalogueoflife.org/ and the Integrated Taxonomic Information System http://www.itis.gov/. Here is a short example using the CoL in which we want to find all the species within the genus *Apis* (honey bees).



                        col_downstream
                        (
                        name 
                        = 
                        "Apis"
                        ٫ 
                        downto 
                        = 
                        "Species"
                        )[[
                        1
                        ]]  

                                                                               
#  childtaxa_id	    childtaxa_name	childtaxa_rank 
# 1	6971712	Apis andreniformis	       Species 
# 2	6971713	       Apis cerana	       Species 
# 3	6971714	      Apis dorsata	       Species 
# 4	6971715	       Apis florea	       Species 
# 5	6971716	Apis koschevnikovi	       Species 
# 6	6845885	    Apis mellifera	       Species 
# 7	6971717	  Apis nigrocincta	       Species 
                    


The result from the above call to *col_downstream()* is a data.frame that gives a number of columns of different information.

### IUCN status

There are a number of things a user can do once they have the correct taxonomic names. One thing a user can do is ask about the conservation status of a species (IUCN Red List of Threatened Species http://www.iucnredlist.org). We have provided a set of functions, *iucn_summary* and *iucn_status*, to search for species names, and extract the status information, respectively. Here, you can search for the panther and lynx.



                        ia 
                        <- iucn_summary
                        (
                        c
                        (
                        "Panthera uncia"
                        ٫ 
                        "Lynx lynx"
                        )) 

                        iucn_status
                        (ia)                                      

                                                                             
# Panthera uncia  Lynx lynx                          
#           "EN"       "LC"                          
                    


It turns out that the panther has a status of endangered (EN) and the lynx has a status of least concern (LC).

### Search for available genes in GenBank

Another use case available in taxize deals with genetic sequences. taxize has three functions to interact with GenBank to search for available genes ( *get_genes_avail*), download genes by GenBank ID ( *get_genes*), and download genes via taxonomic name search, including retrieving a congeneric if the searched taxon does not exist in the database ( *get_seqs*). In this example, one can search for gene sequences for *Umbra limi*.



                        out 
                        <- get_genes_avail
                        (
                        taxon_name 
                        = 
                        "Umbra limi"
                        ٫ 

                                               seqrange 
                        = 
                        "1:2000"
                        ٫       

                                               getrelated 
                        = 
                        FALSE
                        )        
                    


Then one can ask if ’RAG1’ exists in any of the gene names.



                        out[
                        grep
                        (
                        "RAG1"
                        ٫ out$genesavail٫ 
                        ignore.case 
                        = 
                        TRUE
                        )٫ -
                        3
                        ] 

                                                                                  
# 	    spused    length  access_num	      ids 
# 413	Umbra limi	 732	JX190826	394772608 
# 427	Umbra limi	 959	AY459526	 45479841 
# 434	Umbra limi	1631	AY380548	 38858304 
                    


It turns out that there are 430 different unique records found. However, this doesn’t mean that there are 430 different genes found as the API does not provide metadata to classify genes. You can use regular expressions (e.g., *grep*) to search for the gene of interest.

### Matching species tables with different taxonomic resolution

Biologists often need to match different sets of data tied to species. For example, trait-based approaches are a promising tool in ecology ^23^. One problem is that abundance data must be matched with trait databases such as the NCBI Taxonomy database ^24^. These two data tables may contain species information on different taxonomic levels and data might have to be aggregated to a joint taxonomic level, so that the data can be merged. taxize can help in this data-cleaning step, providing a reproducible workflow.

A user can use the mentioned *classification*-function to retrieve the taxonomic hierarchy and then search the hierarchies up- and downwards for matches. Here is an example to match a species (A) with names of on different taxonomic levels (B1 & B2).



                        A 
                        <- 
                        "gammarus roeseli"                                       

                        B1 
                        <- 
                        "gammarus"                                              

                        B2 
                        <- 
                        "gammarus"                                              

                        A_clas 
                        <- classification
                        (A٫ 
                        db
                         = 
                        'ncbi'
                        )                      

                        B1_clas 
                        <- classification
                        (B1٫ 
                        db
                         = 
                        'ncbi'
                        )                    

                        B2_clas 
                        <- classification
                        (B2٫ 
                        db
                         = 
                        'ncbi'
                        )                    

                        A_clas[[
                        1
                        ]]$Rank[
                        tolower
                        (A_clas[[
                        1
                        ]]$ScientificName) %in% B1] 

                                                                                      
# [1] "genus"                                                 
                                                              
A_clas[[
                        1
                        ]]$Rank[
                        tolower
                        (A_clas[[
                        1
                        ]]$ScientificName) %in% B2] 
                                                              
# [1] "family"                                                
                    


If one finds a direct match (here *Gammarus roeseli*), they will be lucky. However, Gammaridae can also be matched with *Gammarus roeseli*, but on a lower taxonomic level. A more comprehensive and realistic example (matching a trait table with an abundance table) is given in Appendix B.

### Aggregating data to a specific taxonomic rank

In biology, one can ask questions at varying taxonomic levels. This use case is easily handled in taxize. A function called *tax_agg* will aggregate community data to a specific taxonomic level. In this example, one can take the data for three species and aggregate them to family level. Again one can specify whether they want to use data from ITIS or NCBI. The rows in the *data.frame* are different communities.



                        data
                        (dune٫ 
                        package 
                        = 
                        'vegan'
                        )                         

                        df 
                        <- 
                        dune[ ٫ 
                        c
                        (
                        1
                        ,
                        3
                        :
                        4
                        )]                               

                        colnames
                        (df) 
                        <- c
                        (
                        "Bellis perennis"
                        ٫
                        "Juncus bufonius"
                        ٫

                                          "Juncus articulatus"
                        )               

                        head
                        (df)                                              

                                                                              
#  Bellis perennis Juncus bufonius Juncus articulatus 
# 2		 3		 0		    0 
# 13		 0		 3		    0 
# 4		 2		 0		    0 
# 16		 0		 0		    3 
# 6		 0		 0		    0 
# 1		 0		 0		    0 
                    




                        agg 
                        <- tax_agg
                        (df٫ 
                        rank
                         = 
                        'family'
                        ٫ 
                        db
                         = 
                        'ncbi'
                        ) 

                        agg                                              
                                                 
#                                                
# Aggregated community data                      
#                                                
# Level of Aggregation: FAMILY                   
# No. taxa before aggregation: 3                 
# No. taxa after aggregation: 2                  
# No. taxa not found: 0                          
                    




                        head
                        (agg$x)                       

                                                          
# 	Asteraceae	Juncaceae 
# 2		 3		0 
# 13		 0		3 
# 4		 2		0 
# 16		 0		3 
# 6		 0		0 
# 1		 0		0 
                    


The two *Juncus* species are aggregated to the family Juncaceae and their abundances are summed. There was only a single species in the family Asteraceae, so the data for *Bellis perennis* are carried over.

## Conclusions

Taxonomic information is increasingly sought by biologists as we take phylogenetic and taxonomic approaches to science. Taxonomic data are becoming more widely available on the web, yet scientists require programmatic access to this data for developing reproducible workflows. taxize was created to bridge this gap - to bring taxonomic data on the web into R, where the data can be easily manipulated, visualized, and analyzed in a reproducible workflow.

We have outlined a suite of use cases in taxize that will likely fit real use cases for many biologists. Of course we have not thought of all possible use cases, so we hope that the biology community can give us feedback on what use cases they want to see available in taxize. One thing we could change in the future is to make functions that fit use cases, and then allow users to select the data source as a parameter in the function. This could possibly make the user interface easier to understand.

taxize is currently under development and will be for some time given the large number of data sources knitted together in the package, and the fact that APIs for each data source can change, requiring changes in taxize code. Contributions to taxize are strongly encouraged, and can be easily done using GitHub here https://github.com/ropensci/taxize_. We hope taxize will be taken up by the community and developed collaboratively, making it progressively better through time as new use cases arise, bug reports are squashed, and contributions are merged.

## Figures and Tables

**APPENDIX A.**
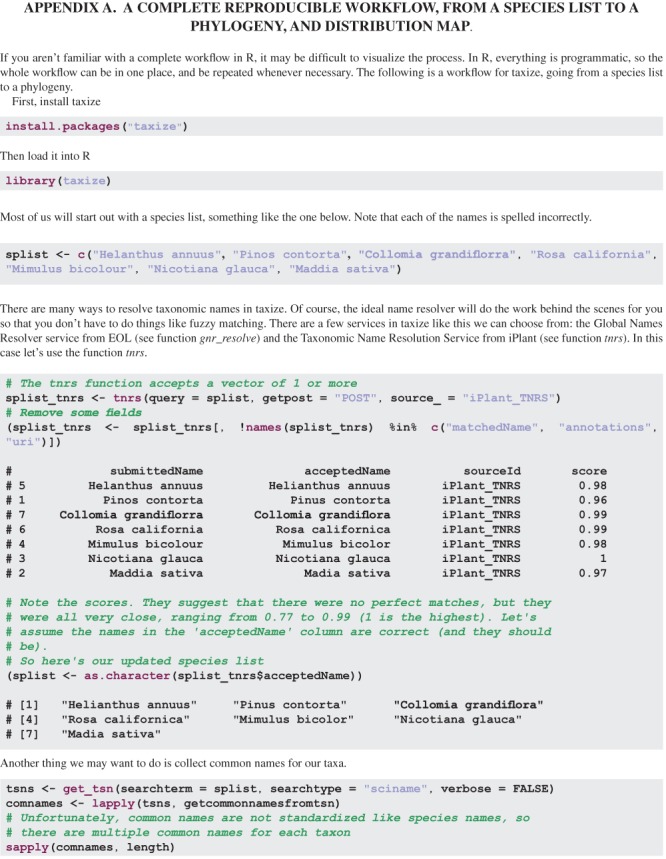
A COMPLETE REPRODUCIBLE WORKFLOW, FROM A SPECIES LIST TO A PHYLOGENY, AND DISTRIBUTION MAP. (Sheet 1)

**APPENDIX A.**
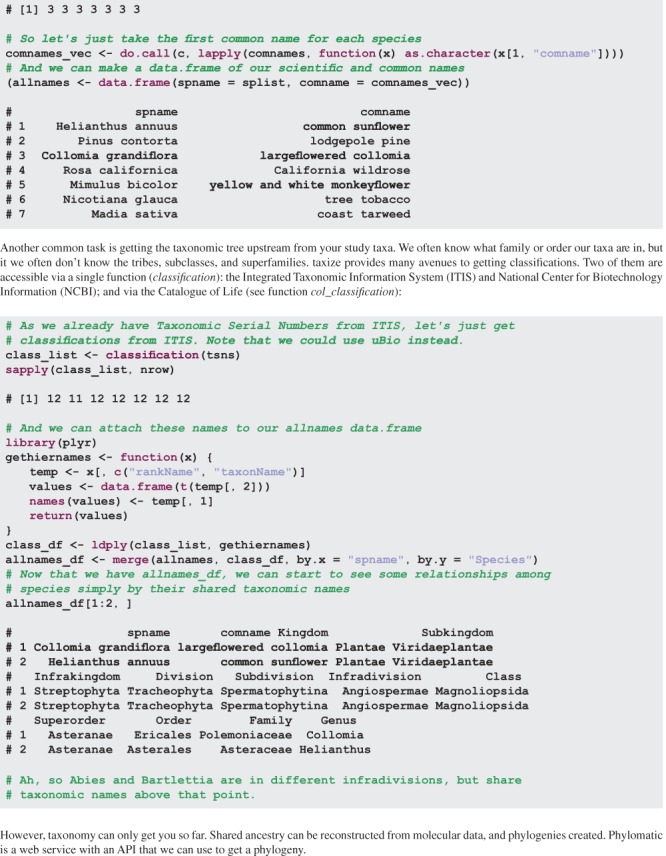
A COMPLETE REPRODUCIBLE WORKFLOW, FROM A SPECIES LIST TO A PHYLOGENY, AND DISTRIBUTION MAP. (Sheet 2)

**APPENDIX A.**
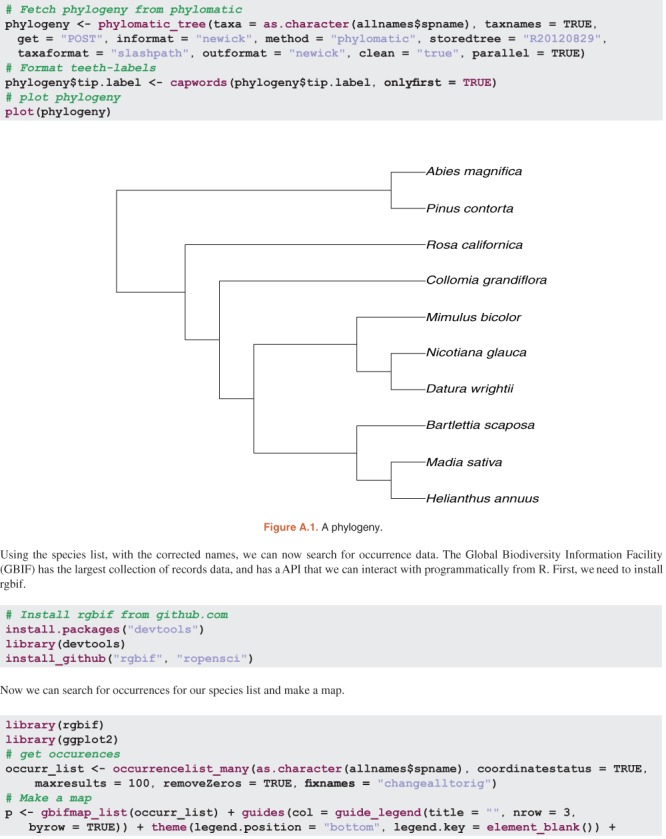
A COMPLETE REPRODUCIBLE WORKFLOW, FROM A SPECIES LIST TO A PHYLOGENY, AND DISTRIBUTION MAP. (Sheet 3)

**APPENDIX A.**
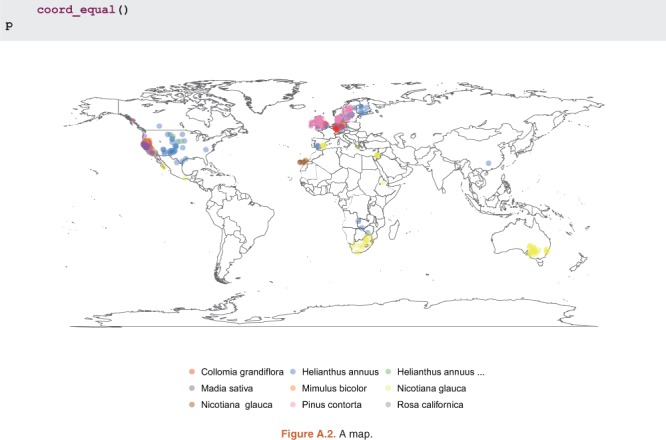
A COMPLETE REPRODUCIBLE WORKFLOW, FROM A SPECIES LIST TO A PHYLOGENY, AND DISTRIBUTION MAP. (Sheet 4)

**APPENDIX B.**
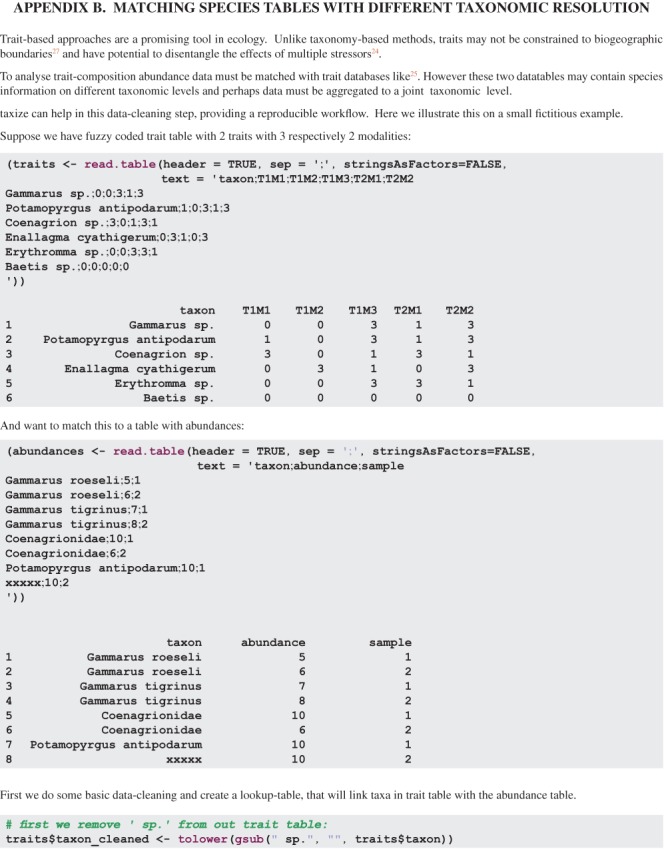
MATCHING SPECIES TABLES WITH DIFFERENT TAXONOMIC RESOLUTION. (Sheet 1)

**APPENDIX B.**
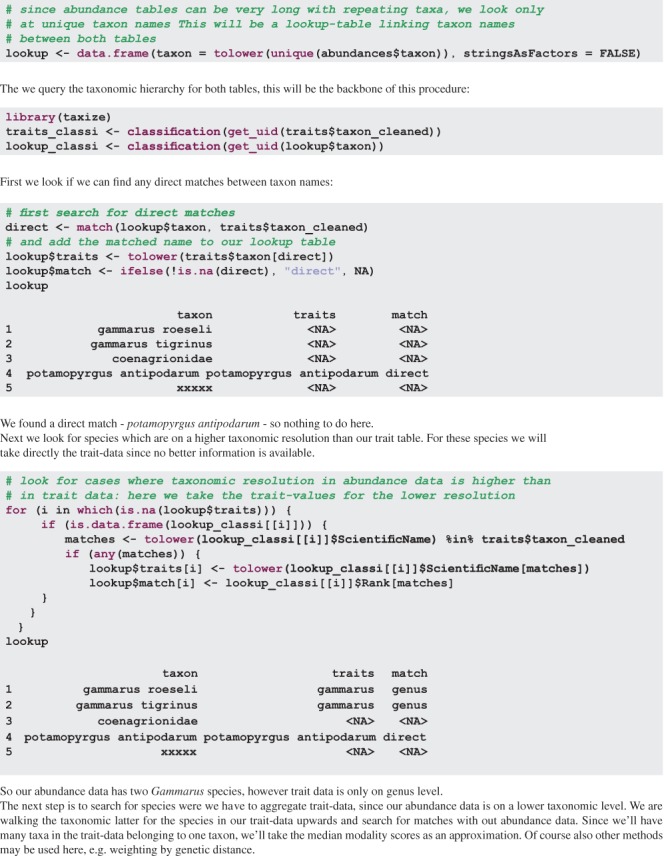
MATCHING SPECIES TABLES WITH DIFFERENT TAXONOMIC RESOLUTION. (Sheet 2)

**APPENDIX B.**
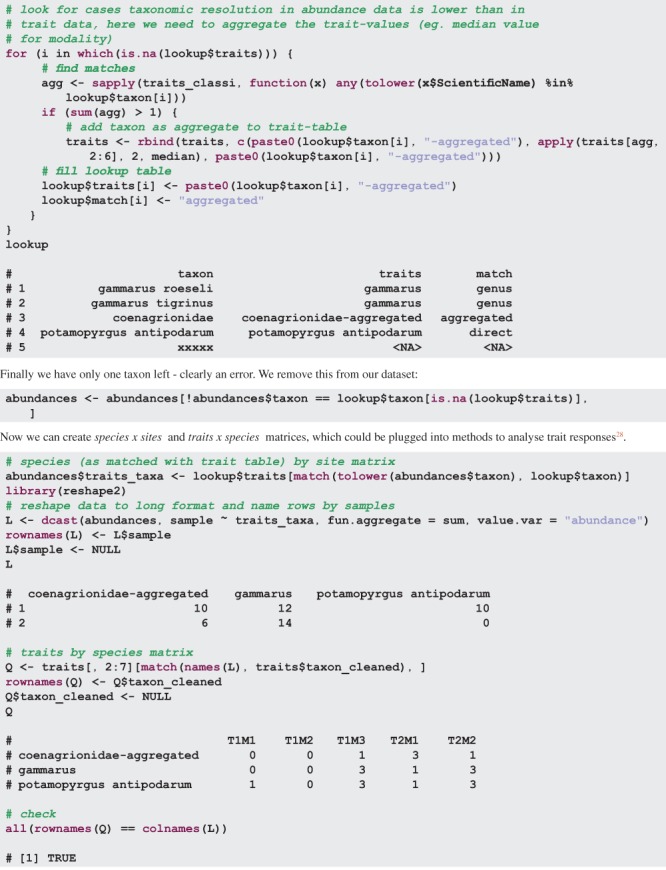
MATCHING SPECIES TABLES WITH DIFFERENT TAXONOMIC RESOLUTION. (Sheet 3)

**APPENDIX B.**
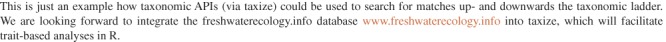
MATCHING SPECIES TABLES WITH DIFFERENT TAXONOMIC RESOLUTION. (Sheet 4)

**APPENDIX C.**
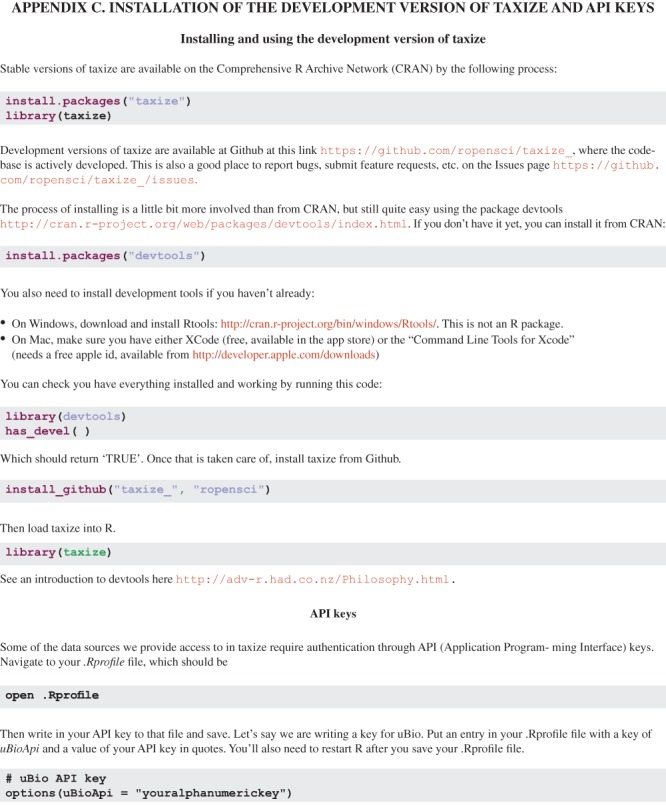
INSTALLATION OF THE DEVELOPMENT VERSION OF TAXIZE AND API KEYS. (Sheet 1)

**APPENDIX C.**
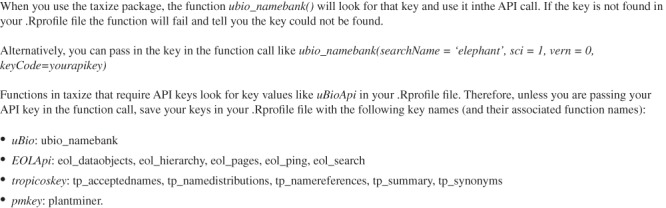
INSTALLATION OF THE DEVELOPMENT VERSION OF TAXIZE AND API KEYS. (Sheet 2)
