# Supplementary material for: taxize: taxonomic search and retrieval in R
Source: F1000Res. 2013 Oct 28;2:191. Originally published 2013 Sep 18. [Version 2] doi: 10.12688/f1000research.2-191.v2 (PMC3901538; doi:10.12688/f1000research.2-191.v2)
Supplement: Peer review history

# Peer review: Referee response for version 2

**Published:** 2013-11-01

**Authors:** Gavin Simpson

**Affiliations:** 1 Department of Biology, University of Regina, Regina, SK, Canada

The author's have addressed my comments on the original version of their manuscript. The issues I pointed to regarding naming conventions were not intended to be addressed now but in a future version of the package. The new appendix is a good addition to the manuscript, although I don't fully follow why "open .Rprofile" is highlighted as code?

I have read this submission. I believe that I have an appropriate level of expertise to confirm that it is of an acceptable scientific standard.

# Response

**Published:** 2013-11-05

**Authors:** Scott Chamberlain

**Affiliations:** Simon Fraser University, Canada

Thanks for the feedback Gavin. Good point that the line "open .Rprofile" should not be highlighted as code. I will see if we can fix that. - Scott

# Peer review: Referee response for version 2

**Published:** 2013-10-29

**Authors:** Will Pearse

**Affiliations:** 1 College of Biological Sciences, University of Minnesota, Minneapolis, MN, USA

The authors have addressed all my concerns, and I think it's perfectly reasonable to address naming conventions in the next release of the package. The third appendix is a particularly nice addition; if the authors intend to alter the manuscript when they next update the package, the section entitled 'API Keys' may contain a minor typo/area that's not perfectly clear (see below) but I was still able to follow the appendix to alter my .Rprofile.

"Navigate to your .Rprofile file, which should be

open .Rprofile"

I have read this submission. I believe that I have an appropriate level of expertise to confirm that it is of an acceptable scientific standard.

# Response

**Published:** 2013-11-05

**Authors:** Scott Chamberlain

**Affiliations:** Simon Fraser University, Canada

Thanks for your comments Will. Gavin mentioned something about the open .Rprofile as well, and we will fix anything there. - Scott

# Peer review: Referee response for version 2

**Published:** 2013-10-28

**Authors:** Ethan White

**Affiliations:** 1 Department of Biology, Utah State University, Logan, UT, USA

The current version of the manuscript addresses all of the recommendations in my previous review. I look forward to seeing the in-progress improvements in the software in its next release.

I have read this submission. I believe that I have an appropriate level of expertise to confirm that it is of an acceptable scientific standard.

# Response

**Published:** 2013-11-05

**Authors:** Scott Chamberlain

**Affiliations:** Simon Fraser University, Canada

Thanks for your constructive comments Ethan. - Scott

# Peer review: Referee response for version 1

**Published:** 2013-09-24

**Authors:** Ethan White

**Affiliations:** 1 Department of Biology, Utah State University, Logan, UT, USA

This software paper describes an R package that provides an integrated R interface for the APIs of over a dozen taxonomically related web services. This is a valuable contribution because it will save researchers time and energy (for those capable of wrapping the APIs themselves), and will allow scientists who lack the technical knowledge to interact with web services themselves to use this data from R. In addition, some of the functions combine existing APIs in useful ways.

The software was developed using version control, on a public development site (https://github.com/ropensci/taxize_), and using a bug tracker. The code is well modularized and includes an extensive test suite. This level of good software practice is notable for scientific software and is indicative of well built and maintained code. It also has a clearly declared CC0 license making it easy for others to use and build on the software.

The software installed easily in R using the standard approach and generally works as expected based on the examples in the paper and on the project's website.

My one major suggestion is to reinforce what the authors' have already suggested in the Conclusions, that it would be an improvement to move to a design that focuses on having a single top-level function for each type of task that the library handles with different data sources being selected using a parameter. This would allow the users to benefit maximally from one of the stronger aspects of this library, which is that it combines access to large numbers of data sources, by making it more of an integrated system and less of a collected set of API wrappers.

Minor issues:

1. The code snippets are images rather than text. This is probably a limitation of the publishing platform, but it does make it more difficult to learn about the software by executing the code snippets.
2. The following two sentences seem rather tangential to the paper and could be removed:
*"Science workflows can now easily incorporate text, code, and images in a single executable document. Reproducible documents should become mainstream in biology to avoid mistakes, and make collaboration easier."*
3. Given that many of the target users of this package will not be particularly familiar with web services and APIs, I would recommend adding another sentence or two to the paragraph on authentication so that readers understand why this is required (i.e., most readers won't understand " *users that abuse the API*") and what it really is (i.e., an individual login of sorts, similar to a username/password).
4. The section on "Aggregating data to a specific taxonomic rank" refers to an example, but none appears to be present.

I have read this submission. I believe that I have an appropriate level of expertise to confirm that it is of an acceptable scientific standard.

# Response

**Published:** 2013-10-14

**Authors:** Scott Chamberlain

**Affiliations:** Simon Fraser University, Canada

We appreciate Dr. White's comments on our manuscript.

We removed the sentences " *Science workflows can now easily incorporate text, code, and images in a single executable document. Reproducible documents should become mainstream in biology to avoid mistakes, and make collaboration easier*."

In response to this reviewer's comment about clarification on APIs and authentication, and Dr. Pearse's comments on the same issue, we have added a new appendix (Appendix C) that explains how to use API keys and install the development version of taxize.

This reviewer commented that the section on Aggregating data to a specific taxonomic rank referred to an example, but none appeared to be present. The example is now in the paper.

# Peer review: Referee response for version 1

**Published:** 2013-09-23

**Authors:** Gavin Simpson

**Affiliations:** 1 Department of Biology, University of Regina, Regina, SK, Canada

Chamberlain and Szöcs present the taxize R package, a set of functions that provides interfaces to several web tools and databases, and simplifies the process of checking, updating, correcting and manipulating taxon names for researchers working with ecological/biological data. A key feature that is repeated throughout is the need for reproducibility of science workflows and taxize provides a means to achieve this within the R software ecosystem for taxonomic search.

The manuscript is well-written and nicely presented, with a good balance of descriptive text and discourse and practical illustration of package usage. A number of examples illustrate the scope of the package, something that is fully expanded upon in the two appendices, which are a welcome addition to the paper.

As to the package, I am not overly fond of long function names; the authors should consider dropping the data source abbreviations from the function names in a future update/revision of the package. Likewise there is some inconsistency in the naming conventions used. For example there is the 'tpl_search()' function to search The Plant List, but the equivalent function to search uBio is 'ubio_namebank()'. Whilst this may reflect specific aspects of terminology in use at the respective data stores, it does not help the user gain familiarity with the package by having them remember inconsistent function names.

One advantage of taxize is that it draws together a rich selection of data stores to query. A further suggestion for a future update would be to add generic function names, that apply to a database connection/information object. The latter would describe the resource the user wants to search and any other required information, such as the API key, etc., for example:

    foo <- taxizeDB(what = "uBio", key = "1646546164694")

The user function to search would then be 'search(foo, "Abies")'. Similar generically named functions would provide the primary user-interface, thus promoting a more consistent toolbox at the R level. This will become increasingly relevant as the scope of taxize increases through the addition of new data stores that the package can access.

In terms of presentation in the paper, I really don't like the way the R code inputs merge with the R outputs. I know the author of Knitr doesn't like the demarcation of output being polluted by the R prompt, but I do find it difficult parsing the inputs/outputs you show because often there is no space between them and users not familiar with R will have greater difficulties than I. Consider adding in more conventional indications of R outputs, or physically separate input from output by breaking up the chunks of code to have whitespace between the grey-background chunks. Related, in one location I noticed something amiss with the layout; in the first code block at the top of page 5, the printed output looks wrong here. I would expect the attributes to print on their own line and the data in the attribute to also be on its own separate line.

Note also, the inconsistency in the naming of the output object columns. For example, in the two code chunks shown in column 1 of page 4, the first block has an object printed with column names 'matched_name' and 'data_source_title', whilst camelCase is used in the outputs shown in the second block. As the package is revised and developed, consider this and other aspects of providing a consistent presentation to the user.

I was a little confused about the example in the section Resolve Taxonomic Names on page 4. Should the taxon name be " *Helianthus annuus*" or " *Helianthus annus*"? In the 'mynames' definition you include ' *Helianthus annuus*' in the character vector but the output shown suggests that the submitted name was ' *Helianthus annus*' (1 "u") in rows with rownames 9 and 10 in the output shown.

Other than that there were the following minor observations:

1. Abstract: replace "easy" with "simple" in "... *fashion that's easy*...", and move the details about availability and the URI to the end of the sentence.
2. Page 2, Column 1, Paragraph 2: You have " *I* *n addition, there is no one authoritative taxonomic names source*...", which is a little clumsy to read. How about " *In addition, there is no one authoritative source of  taxonomic names*..."?
3. Pg 2, C1, P2-3: The abbreviated data sources are presented first (in paragraph 2) and subsequently defined (in para 3). Restructure this so that the abbreviated forms are explained upon first usage.
4. Pg 2, C2, P2: Most R packages are "in development" so I would drop the qualifier and reword the opening sentence of the paragraph.
5. Pg 2, C2, P6: Change " *and more can easily be added*" to " *and more can be easily added*" seems to flow better?
6. Pg 5, paragraph above Figure 1: You refer to converting the object to an **ape** *phylo* object and then repeat essentially the same information in the next sentence. Remove the repetition.
7. Pg 6, C1: The header may be better as " *Which taxa are children of the taxon of interest*".
8. Pg 6: In the section "IUCN status", the term "we" is used to refer to both the authors and the user. This is confusing. Reserve "we" for reference to the authors and use something else ("a user" perhaps) for the other instances. Check this throughout the entire manuscript.
9. Pg 6, C2: in the paragraph immediately below the 'grep()' for "RAG1", two consecutive sentences begin with "However".
10. Pg 7: The first sentence of "Aggregating data...." reads " *In biology, one can asks questions*...". It should be " *one ask*s" or " *one can ask*".
11. Pg 7, Conclusions: The first sentence reads *"* *information is increasingly sought out by biologists*". I would drop "out" as "sought" is sufficient on its own.
12. Appendices: Should the two figures in the Appendices have a different reference to differentiate them from Figure 1 in the main body of the paper? As it stands, the paper has two Figure 1s, one on page 5 and a second on page 12 in the Appendix.
13. On Appendix Figure 2: The individual points are a little large. Consider reducing the plotting character size. I appreciate the effect you were going for with the transparency indicating density of observation through overplotting, but the effect is weakened by the size of the individual points.
14. Should the phylogenetic trees have some scale to them? I presume the height of the stems is an indication of phylogenetic distance but the figure is hard to calibrate without an associated scale. A quick look at Paradis (2012) Analysis of Phylogenetics and Evolution with R would suggest however that a scale is not consistently applied to these trees. I am happy to be guided by the authors as they will be more familiar with the conventions than I.

I have read this submission. I believe that I have an appropriate level of expertise to confirm that it is of an acceptable scientific standard.

# Response

**Published:** 2013-10-14

**Authors:** Scott Chamberlain

**Affiliations:** Simon Fraser University, Canada

We appreciate Dr. Simpson's very thorough comments on our manuscript. The following are responses to Dr. Simpson's comments:

*- ...there is some inconsistency in the naming conventions used. For example there is the 'tpl_search()' function to search The Plant List, but the equivalent function to search uBio is 'ubio_namebank()'. Whilst this may reflect specific aspects of terminology in use at the respective data stores, it does not help the user gain familiarity with the package by having them remember inconsistent function names.*

We agree that we should definitely improve naming conventions for functions. However, we think it's better to change the function names as needed in an upcoming version of the software after we have had time work on the problem.

*- Consider adding in more conventional indications of R outputs, or physically separate input from output by breaking up the chunks of code to have whitespace between the grey-background chunks.*

We have used comments (pound signs) for the results of function calls within each code block to indicate output as separate from code input. This way users can copy/paste code directly into R to try it out.

*- in one location I noticed something amiss with the layout; in the first code block at the top of page 5, the printed output looks wrong here. I would expect the attributes to print on their own line and the data in the attribute to also be on its own separate line.*

This was a problem with the typesetting, and we have fixed it.

*- the inconsistency in the naming of the output object columns. For example, in the two code chunks shown in column 1 of page 4, the first block has an object printed with column names 'matched_name' and 'data_source_title', whilst camelCase is used in the outputs shown in the second block.*

We agree that we should definitely improve naming conventions for object columns. However, we think it's better to change the column names as needed in an upcoming version of the software after we have had time work on the problem.

*- I was a little confused about the example in the section Resolve Taxonomic Names on page 4. Should the taxon name be "Helianthus annuus" or "Helianthus annus"? In the 'mynames' definition you include 'Helianthus annuus' in the character vector but the output shown suggests that the submitted name was 'Helianthus annus' (1 "u") in rows with rownames 9 and 10 in the output shown.*

**Fixed.**

*- Abstract: replace "easy" with "simple" in "...fashion that's easy...", and move the details about availability and the URI to the end of the sentence.*

**Fixed.**

*- Page 2, Column 1, Paragraph 2: You have "In addition, there is no one authoritative taxonomic names source...", which is a little clumsy to read. How about "In addition, there is no one authoritative source of taxonomic names..."?*

**Changed.**

*- Pg 2, C1, P2-3: The abbreviated data sources are presented first (in paragraph 2) and subsequently defined (in para 3). Restructure this so that the abbreviated forms are explained upon first usage.*

**Changed.**

*- Pg 2, C2, P2: Most R packages are "in development" so I would drop the qualifier and reword the opening sentence of the paragraph.*

**Changed.**

*- Pg 2, C2, P6: Change "and more can easily be added" to "and more can be easily added" seems to flow better?*

**Changed.**

*- Pg 5, paragraph above Figure 1: You refer to converting the object to an ape phylo object and then repeat essentially the same information in the next sentence. Remove the repetition.*

**Removed.**

*- Pg 6, C1: The header may be better as "Which taxa are children of the taxon of interest".*

**Changed.**

*- Pg 6: In the section "IUCN status", the term "we" is used to refer to both the authors and the user. This is confusing. Reserve "we" for reference to the authors and use something else ("a user" perhaps) for the other instances. Check this throughout the entire manuscript.*

**Fixed.**

*- Pg 6, C2: in the paragraph immediately below the 'grep()' for "RAG1", two consecutive sentences begin with "However".*

**Changed.**

*- Pg 7: The first sentence of "Aggregating data...." reads "In biology, one can asks questions...". It should be "one asks" or "one can ask"*

**Changed.**

*- Pg 7, Conclusions: The first sentence reads "information is increasingly sought out by biologists". I would drop "out" as "sought" is sufficient on its own.*

**Changed.**

*- Appendices: Should the two figures in the Appendices have a different reference to differentiate them from Figure 1 in the main body of the paper? As it stands, the paper has two Figure 1s, one on page 5 and a second on page 12 in the Appendix.*

**Fixed.**

*- On Appendix Figure 2: The individual points are a little large. Consider reducing the plotting character size. I appreciate the effect you were going for with the transparency indicating density of observation through overplotting, but the effect is weakened by the size of the individual points*.

Although we agree that the styling of the figure could be improved, we are going to leave it as is because it's not important for understanding the material.

*- Should the phylogenetic trees have some scale to them? I presume the height of the stems is an indication of phylogenetic distance but the figure is hard to calibrate without an associated scale. A quick look at Paradis (2012) Analysis of Phylogenetics and Evolution with R would suggest however that a scale is not consistently applied to these trees. I am happy to be guided by the authors as they will be more familiar with the conventions than I.*

A scale could be used for sure. However, our focus is on showing readers that they can get data which can be used to make a phylogeny, not on how to properly create and display a phylogeny. Thus, we are leaving the phylogeny as is without a scale.

# Peer review: Referee response for version 1

**Published:** 2013-09-19

**Authors:** Will Pearse

**Affiliations:** 1 College of Biological Sciences, University of Minnesota, Minneapolis, MN, USA

The software this article describes is well-written and of use to ecologists. The guide and appendices in this article give a good overview of the features of the package, and are well-written. The title and abstract are well-written.

My only serious comment would be that the authors refer to species' taxonomy as if it perfectly reflects species' phylogeny (e.g. the end of the first paragraph in the introduction and the first paragraph in the section "retrieve higher taxonomic names"). More often than not, taxonomy does reflect phylogeny, but a sentence clarifying this distinction somewhere might be helpful.

A few minor things:

- Table 1 - "National Center for Biotechnology Information Federhen" - the author's surname, but not the citation, is in the text; "searchbycommonname" and "searchbyscientificname" seem to be the only elements not in alphabetical order in the table.

- Phylomatic can now create phylogenies for mammals, not just angiosperms (e.g. "retrieve a phylogeny").

- Some sentences could perhaps be linked more neatly in the introduction (e.g. " *gives that name as unresolved. But Helianthus*", " *moved to the cloud. Therefore there is a need*", " *additional confusion. Last, name*"). Similarly, page 3 has some very short paragraphs; could the descriptions of *taxonstand* and The Plant List be moved to table 1? "Science workflows" or "scientific workflows" (fourth paragraph of introduction)? These are all very minor points!

- Perhaps an example of what to type in order to add API keys into the .Rprofile would be helpful, or maybe could just be added to the help file for " *ubio_namebank*" as this is the function named when it's mentioned.

I have read this submission. I believe that I have an appropriate level of expertise to confirm that it is of an acceptable scientific standard.

# Response

**Published:** 2013-10-14

**Authors:** Scott Chamberlain

**Affiliations:** Simon Fraser University, Canada

We appreciate Dr. Pearse's comments on our manuscript. We agree that species taxonomy does not equate to phylogenetic history, so we added the following sentence to the first paragraph: *"Although taxonomic classifications are human constructs created to understand the real phylogeny of life \cite{benton2000}, they are nonetheless essential to organize the vast diversity of organisms*." We fixed the citation in Table 1, and reordered the functions in the table so as to be alphabetical. Thanks for pointing out that Phylomatic now accepts mammals in addition to Angiosperm plants - we have adjusted the language accordingly.

We removed the description of Taxonstand and the Plantlist.org (and associated references) that this reviewer referred to as it wasn't necessary and improves reading.

This reviewer asked for a better explanation of how to use the API keys and the .Rprofile file. In response, we have added a new appendix (Appendix C) that explains using API keys and installing the development version of taxize.
